# Assessment of combined use of ArcCheck^®^ detector and portal dosimetry for delivery quality assurance of head and neck and prostate volumetric‐modulated arc therapy

**DOI:** 10.1002/acm2.12460

**Published:** 2018-10-19

**Authors:** Gilles Moliner, Lise Sorro, Rodolfe Verstraet, Paul Alexandre Daviau, Mélanie Casas, Bérengère Piron, Karine Dubois, Charles Debrigode, Corinne Barrau, Françoise Bons, Joël Greffier

**Affiliations:** ^1^ Department of Radiotherapy Nîmes University Hospital Nîmes Cedex France; ^2^ Medical Physics Unit Nîmes University Hospital Nîmes Cedex France

**Keywords:** ArcCheck, Gamma index, PDIP, VMAT pretreatment quality assurance

## Abstract

**Purpose:**

To assess the efficiency of combined use of ArcCheck^®^ detector (AC) and portal dosimetry (PDIP) for delivery quality assurance of head and neck and prostate volumetric‐modulated arc therapy.

**Materials and methods:**

Measurement processes were studied with the Gamma index method according to three analysis protocols. The detection sensitivity to technical errors of each individual or combined measurement processes was studied by inserting collimator, dose and MLC opening error into five head and neck and five prostate initial treatment plans. A total of 220 plans were created and 660 analyses were conducted by comparing measurements to error free planned dose matrix.

**Results:**

For head and neck localization, collimator errors could be detected from 2° for AC and 3° for PDIP. Dose and MLC errors could be detected from 2% and 0.5 mm for AC and PDIP. Depending on the analysis protocol, the detection sensitivity of total simulated errors ranged from 54% to 88% for AC vs 40% to 74% for PDIP and 58% to 92% for the combined process. For the prostate localization, collimator errors could be detected from 4° for AC while they could not be detected by PDIP. Dose and MLC errors could be detected from 3% and 0.5 mm for AC and PDIP. The detection sensitivity of total simulated errors ranged from 30% to 56% for AC vs 16% to 38% for PDIP and 30% to 58% for combined process.

**Conclusion:**

The combined use of the two measurement processes did not statistically improve the detectability of technical errors compared to use of single process.

## 1 INTRODUCTION

Volumetric‐modulated arc therapy (VMAT) has become widely used, especially for head and neck (H&N) and prostate cancer treatments. VMAT dose distribution depends on the dose rate modulation, arm movement speed, multi‐leaf collimator (MLC) position, and collimator angulation.^1^ The large number of degrees of technical freedom allows a significant preservation of organs at risk.^2^, ^3^, ^4^, ^5^ However, the complexity of this technique requires verification of the planned dose distribution by performing quality control (QC) treatment plans. These QCs are performed prior to treatment and consist of checking the concordance between planned dose distribution and real distribution delivered by the linear accelerator. Different measurement systems are available: the electronic portal imaging device (EPID), 2‐D or semi 3‐D independent detector, or ionization chamber. The dose distribution analysis is generally performed with the Gamma index method with highly versatile analysis protocols regarding the choice of the deviation criterion of dose and acceptable distance, the selection of the pixels to be analyzed, the normalization method, and the Gamma pass rate criterion.^6^, ^7^, ^8^, ^9^ Depending on the experience curve acquired by the teams, these different verification methods can be used individually or in combination, which can make the pretreatment verification process very time‐consuming. In this context, the purpose of this study was to determine the efficiency of combined use of ArcCheck^®^ detector (Sun Nuclear, Melbourne, FL, USA) and portal dosimetry with EPID As 1200^®^ (Varian Medical System, Palo Alto, CA, USA) for H&N and prostate VMAT delivery quality assurance. The detectability threshold of both measurement processes to different types of technical errors was studied using the Gamma index method according to three analysis protocols. Then, the error detection sensitivity of each individual and combined measurement process was compared.

## 2 MATERIALS AND METHODS

### 2.A Measurement processes

Measurements were made on a Truebeam^®^ 2.0 linear accelerator (Varian Medical System, Palo Alto, CA, USA) between January 2017 and May 2017.

Three measurement processes were assessed:

- ArcCheck^®^ detection phantom associated with SNC patient™ software v6.7.1 (AC)
- EPID As 1200^®^ associated with Portal Dose Image Prediction PDIP^®^ v13.0 (PDIP)
- Combination of AC and PDIP which consisted of accumulating the verification information of the two systems (Combined).

### 2.A.1 ArcCheck^®^

The ArcCheck^®^ detector is a cylindrical acrylic phantom made up of 1386 diodes arranged at a depth of 2.9 cm and positioned 1 cm apart. Its length, external, and internal diameter are, respectively, 21.0, 26.6, and 15.1 cm. The active area of each diode is 0.8 × 0.8 mm². Combined with the SNC patient™ v6.7.1 software, it provides absolute or relative semi‐3‐D dose distribution measurements. Before each measurement session, a background correction and a 10 × 10 cm² open field, source axis distance (SAD) 100 cm, was delivered and used for dose calibration following the manufacturer's recommendation.

### 2.A.2 EPID As 1200^®^

The EPID As 1200^®^ is a 43 × 43 cm² Amorphous Silicon (A‐Si) semiconductor active matrix of area which consists of 1280 × 1280 pixels of 0.34 × 0.34 mm² for 2‐D distribution measurement. Associated with the PDIP^®^ v13.0 module, this detector does not provide an absolute dose measurement but gives a signal in Calibration Unit (CU). EPID calibration was performed before the first measurement session. Dark field and flood field calibrations were performed before a 10 × 10 cm² open field, SAD 100 cm, used for dose calibration according to the manufacturer's protocol. Calibration verification was performed before each measurement session.

### 2.B Treatment planning and pretreatment QC preparation

Treatment planning for H&N and prostate localizations were performed with Eclipse^®^ v13.5 software (Varian Medical System, Palo Alto, CA, USA). For prostate treatment plans, a prescribed dose of 76 Gy with a fraction of 2 Gy was delivered. For H&N treatment plans, the prescribed dose ranged from 66 to 70 Gy (three dose levels corresponding to high‐risk CTV (66–70 Gy), intermediate risk CTV (57–60 Gy), and low‐risk CTV (54–56 Gy)) with a fraction of 2–2.12 Gy. For each localization, the plan was delivered by two coplanar arcs, one clockwise, and one counterclockwise, with a 6 MV energy and a maximum dose rate of 600 UM/min which are the parameters usually used in our institution.

**Figure 1:**
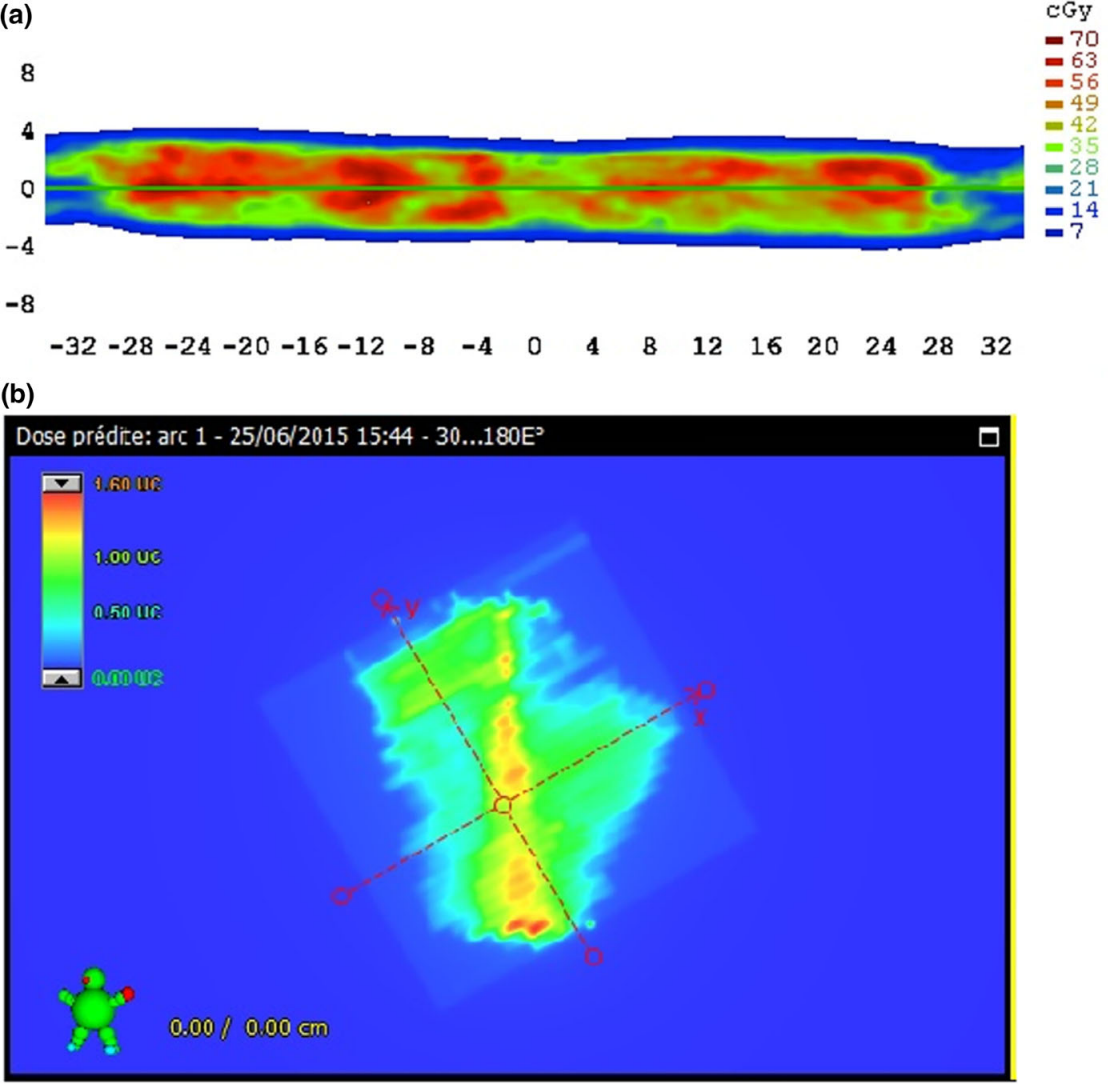
Calculated dose matrix in Eclipse v13.5 software. Computed dose matrix in AC (a) and EPID (b).

For both AC and PDIP processes, the treatment plan was computed in the ArcCheck^®^ or EPID As 1200^®^ image to obtain the planned dose or CU matrix (Fig. 1).

### 2.C Dose matrix concordance analysis

Planned and delivered dose matrix comparisons were performed using the Gamma index method proposed in 1998 by Low et al.^10^ Three combinations of parameters for the Gamma index found in the literature were used and are detailed in Table 1.^11^, ^12^ The dose difference criteria (DD) and the distance to agreement criteria (DTA) match, respectively, the difference in dose and distance accepted. Global and local mode of dose normalization (Mode) were studied. The threshold pixels criterion (TH) in terms of percentage of the maximum dose for AC and area of the MLC complete irradiated area outline (CIAO) for PDIP was evaluated. Thresholding of all the pixels was made at a dose greater than or equal to 10% (10% Dmax) and 20% (20% Dmax) of the maximum dose for AC. For PDIP, we used our historical threshold criteria MLC CIAO + 1 cm which corresponds to the opening envelop of the MLC incremented by 1 cm. The passing rate (PR) for the plan to be considered to conform corresponds to the minimum percentage of pixels having a Gamma index less than one (GI <1).

**Table 1:** Concordance analysis protocol for planned and measured dose matrices. DD is the accepted dose difference. DTA is the distance difference accepted. Mode corresponds to the dose normalization mode. TH corresponds to the thresholding pixel criterion. 10% Dmax and 20% Dmax correspond to a thresholding of all the pixels, with a dose greater than or equal to 10% and 20% of the maximum dose of the plan. MLC CIAO + 1 cm correspond to a threshold of all the pixels included in Complete Irradiated Area Outline (CIAO) of the MLC incremented by 1 cm. PR is the minimum success criterion on the pixel percentage with a Gamma index less than one for the plan to be considered compliant

Protocol DD/DTA/Mode	Process	TH	PR
3%/3 mm/Global	AC	10% Dmax	95%
	PDIP	MLC CIAO +1 cm	
3%/3 mm/Local	AC	20% Dmax	95%
	PDIP	MLC CIAO +1 cm	
2%/2 mm/Global	AC	10% Dmax	90%
	PDIP	MLC CIAO +1 cm	

### 2.D Assessment of process sensitivity for various potential technical errors

To assess the detection sensitivity of AC and PDIP processes, the following potential technical errors were simulated:

- Collimator error (from 1° to 5°, increment of 1°)
- Dose error (equal to +2% and +3%)
- MLC opening error (equal to +0.5 mm, +1 mm and +2 mm)

These errors were inserted into five H&N and five prostate initial DICOM‐RT treatment plans using a MATLAB 2017^®^ routine (The MathWorks, Natick, MA, USA). A total of 10 error‐free plans and 100 plans with errors were respectively computed for AC and then for PDIP (50 plans with a collimator error, 20 plans with a dose error, and 30 plans with an MLC opening error). The comparison of all the measurements was made with the error‐free planned dose matrix. In total, 220 plans were computed and 660 analyses were performed. The average detection threshold of each type of error was determined for both measurement processes as follows: mean PR for each type and level of error were calculated. If the mean PR was below the protocol tolerance limit studied, then the level and type of error could be detected in agreement with the AAPM report TG‐218 which reports the risk of systematic errors when the plan does not pass the tolerance limit.^9^ The error detection sensitivity by type and cumulative was also calculated for each individual process (AC or PDIP) (*δ*Error_*individual process*_) and for the combined measurement process (AC + PDIP) (*δ*Error_*combined processes*_) according to formulae 1 and 2. The error was considered detected when the plan no longer respected the PR. For the combined process, we cumulated all errors detected by at least one of the two systems.

(1) $$\begin{array}{c} \delta {\text{Error}}_{individual\;process}\left(\%\right)=\left(Total\;plans\;with\;errors\;detected\;by\;process\;Total\;plans\;with\;errors\right)\times 100 \end{array}$$

(2) $$\begin{array}{c} \delta {\text{Error}}_{combined\;processes}\left(\%\right)=\left(Total\;plans\;with\;errors\;detected\;by\;at\;least\;one\;process/Total\;plans\;with\;errors\right)\times 100 \end{array}$$

The statistical comparison of error detection sensitivity was performed by Chi‐squared test and a *P*‐value <0.05 was considered statistically significant.

## 3 RESULTS

**Figure 2:**
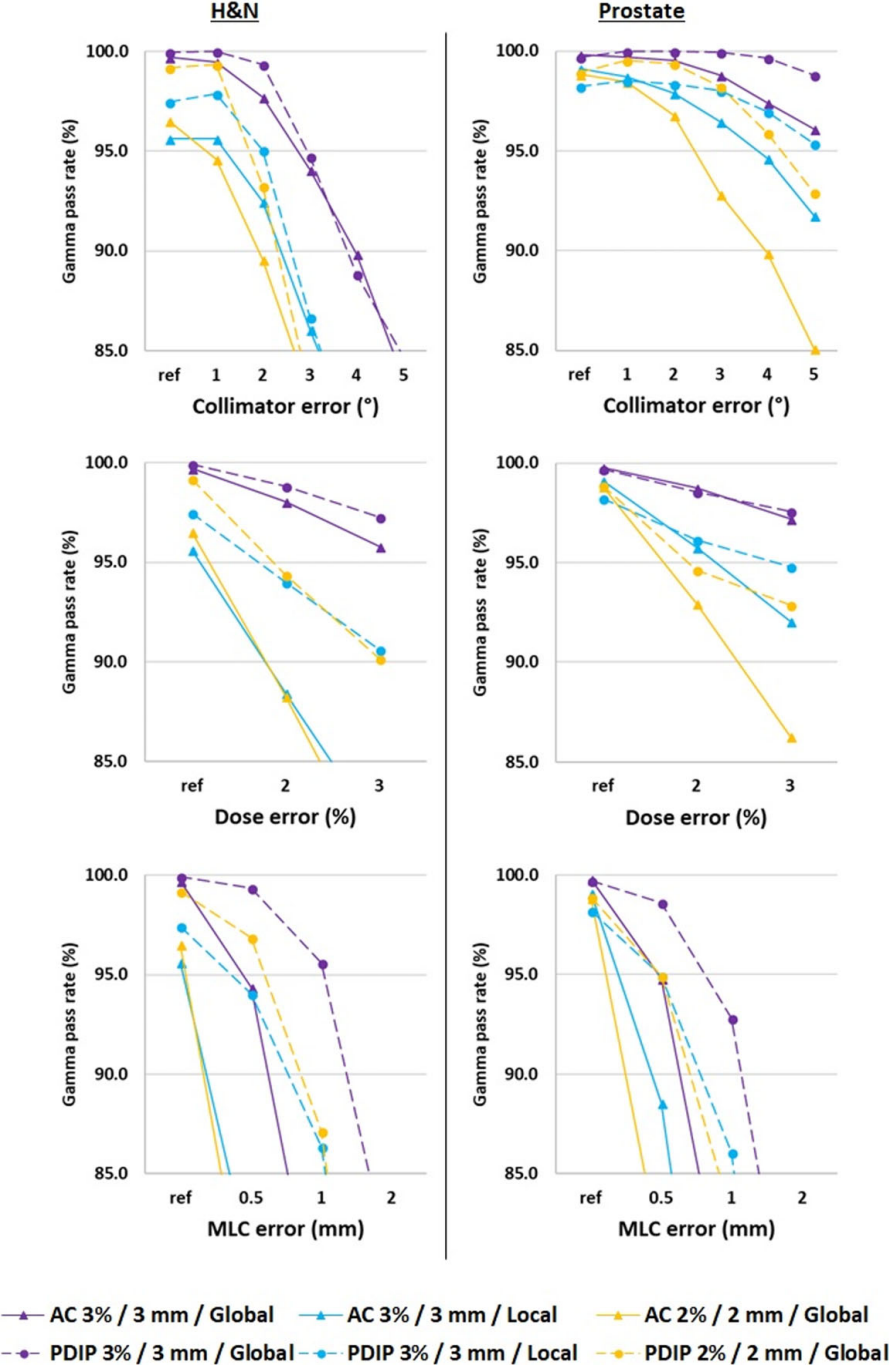
Average percentage of pixels with a gamma index less than one as a function of the error free plans (ref) and the different types of errors simulated for the head and neck (H&N) and prostate localizations.

Dose calibration verification for AC and PDIP showed a discrepancy between the calculated and measured dose at the isocenter less than 0.5%. The mean PR of reference plans (ref) and plans with collimator, dose, and MLC errors for AC and PDIP processes according to the three Gamma index protocols and localization are presented in Fig. 2. For H&N localization, collimator errors could be detected from 2° for AC (mean PR ± SD = 92.5% ± 2.9%) and 3° for PDIP (86.7% ± 6.7%) with 3%/3 mm/Local analysis protocol. Dose errors could be detected from 2% for AC with 3%/3 mm/Local (88.5% ± 5.1%) and 2%/2 mm/Global (88.3% ± 6.3%) analysis protocol. PDIP could detect dose errors from 2% (94% ± 1.7%) only with 3%/3 mm/Local analysis protocol. MLC errors could be detected from 0.5 mm for AC with all analysis protocols. PDIP could detect these errors from 0.5 mm (94% ± 3%) only with 3%/3 mm/Local analysis protocol. Similarly, for the prostate localization, collimator errors could be detected from 4° for AC with 3%/3 mm/Local (94.6% ± 2.3%), and 2%/2 mm/Global (89.9% ± 3.3%) analysis protocol while they could not be detected by PDIP. Dose errors could be detected from 3% for AC with 3%/3 mm/Local (92.1% ± 2.5%) and 2%/2 mm/Global (86.3% ± 5.8%) analysis protocol. PDIP could detect dose errors from 3% (94.8% ± 2%) only with 3%/3 mm/Local analysis protocol. MLC errors could be detected from 0.5 mm for AC with all analysis protocol. PDIP could detect these errors from 0.5 mm (94.9% ± 2.9%) only with 3%/3 mm/Local analysis protocol.

For ref H&N plans, mean PR varied from 99.7% ± 0.4% to 95.6% ± 2.6% for AC and 99.9% ± 0.1% to 97.5% ± 0.9% for PDIP depending on analysis protocol. Similarly, for ref Prostate plans, mean PR varied from 99.8% ± 0.2% to 98.8% ± 0.8% for AC and 99.7% ± 0.4% to 98.2% ± 2.6%.

Tables 2 and 3 present the error detection sensitivity according to the three Gamma index protocols for AC, PDIP, and combined processes for H&N and prostate treatment plans. For H&N localization, by varying the analysis protocol from 3%/3 mm/Global to 3%/3 mm/Local, the detection sensitivity of total simulated errors ranged from 54% to 88% for AC vs 40% to 74% for PDIP and 58% to 92% for combined process (Table 2).

**Table 2:** Error detection sensitivity for AC, PDIP and combined processes with three settings of Gamma index for head and neck (H&N) treatment plans. *δ*Total, *δ*Colli, *δ*Dose, and *δ*MLC correspond to the detection sensitivity of total error, collimator angulation error, dose error, and MLC error

Protocol	Process	*δ*Total(%)	*δ*Colli(%)	*δ*Dose(%)	*δ*MLC(%)
3%/3 mm/Global	AC	54	44	30	87
	PDIP	40	48	10	47
	Combined	58	52	30	87
3%/3 mm/Local	AC	88	80	90	100
	PDIP	74	60	90	87
	Combined	92	84	100	100
2%/2 mm/Global	AC	76	68	70	93
	PDIP	50	52	30	60
	Combined	80	72	80	93

**Table 3:** Error detection sensitivity for AC, PDIP, and combined processes with three settings of Gamma index for prostate treatment plans. *δ*Total, *δ*Colli, *δ*Dose, and *δ*MLC correspond to the detection sensitivity of total error, collimator angulation error, dose error, and MLC error

Protocol	Process	*δ*Total(%)	*δ*Colli(%)	*δ*Dose(%)	*δ*MLC(%)
3%/3 mm/Global	AC	30	8	10	80
	PDIP	16	0	0	53
	Combined	30	8	10	80
3%/3 mm/Local	AC	56	28	60	100
	PDIP	38	16	40	73
	Combined	58	28	70	100
2%/2 mm/Global	AC	52	28	50	93
	PDIP	24	0	30	60
	Combined	56	28	70	93

For prostate localization, this rate ranged from 30% to 56% for AC vs 16% to 38% for PDIP and 30% to 58% for combined process (Table 3).

Regardless of the measurement process, the H&N error detection sensitivity was statistically superior compared to prostate (*P*‐value <0.01).

Irrespective of the localization, AC with 3%/3 mm/Local analysis protocol could detect all the MLC errors plans. Moreover, AC allowed a significant improvement of error detection sensitivity compared to PDIP (*P*‐value <0.01). Regardless of the analysis protocol used, the combined measurement process did not statistically improve errors detection sensitivity compared to AC for H&N (*P*‐value = 0.26) and prostate (*P*‐value = 0.62). PDIP 3%/3 mm/Local analysis protocol did statistically improve errors detection sensitivity compared to combined process with 3%/3 mm/Global analysis protocol for H&N (*P*‐value = 0.01) and was equivalent for prostate (*P*‐value = 0.23). Similarly, AC 3%/3 mm/Local analysis protocol did statistically improve errors detection sensitivity compared to combined process with 3%/3 mm/Global analysis protocol for H&N (*P*‐value <0.001) and prostate (*P*‐value <0.001).

## 4 DISCUSSION

VMAT pretreatment delivery quality assurance is an essential step before final approval of the treatment plan. Depending on the verification process protocol, this step can be very time‐consuming. Previous studies have evaluated the sensitivity of detection of potential technical errors by different measurement processes and Gamma analysis protocols.^11^, ^12^, ^13^, ^14^, ^15^ The goal of this study was to assess the efficiency of combined use of AC and PDIP for H&N and prostate VMAT delivery quality assurance.

Regardless of the localization, the mean detection threshold of errors appeared equivalent between the two measurement processes with the optimal analysis protocol. Only the collimator error could be detected earlier from 2° for the H&N localization and 4° for the prostate localization by the AC (Fig. 2). The results of this study concerning the detection threshold of simulated technical errors seem consistent with the litterature, since Vieillevigne et al. found a detection threshold for a 2%/2 mm/Global analysis protocol with AC from 2° for collimator errors and 0.5 mm for MLC errors.^11^ Fredh et al. showed a late detection of collimator errors for brain, and prostate treatment plans by different measuring systems.^13^ Steer et al. also failed to detect dose errors less than or equal to 3% when using a Gamma index protocol 3%/3 mm/Global.^14^

Concerning the error detection sensitivity, with an equivalent analysis protocol, the AC was higher than PDIP irrespective of the localization studied. AC detected 76% to 88% of total errors by varying analysis protocol from 2%/2 mm/Global to 3%/3 mm/Local for H&N and 52% to 56% for Prostate. Similarly, PDIP detected 50% to 74% of total errors for H&N and 24% to 38% for prostate. These results differ from Fredh et al. where EPID based EPIQA™ (EPIDos, Bratislava, Slovak Republic) detected all simulated errors (20/20) compared to Delta4^®^ phantom (Scandidos AB, Uppsala, Sweden) which detected 15 of 20 errors with 2%/2 mm/Global/TH 10% analysis protocol^13^ and with Bruschi et al. who demonstrated that the detection of delivered errors was improved with the increase of detector spatial resolution by comparing planar detector array with different spatial resolution.^15^ Vieillevigne et al. showed that for a 2%/2 mm/Global/TH 10% protocol, EPIQA™ detected 14 errors out of 20 vs 12 errors for AC.^11^ These differences can be explained by the shape of the detection matrix (Delta4^®^ vs AC), the measurement method, the dose calculation algorithm (PDIP vs EPIQA™), as well as by the threshold criterion used (MLC CIAO + 1 cm vs TH 10%). Indeed, the comparison of AC and PDIP with a different thresholding criterion is a limitation of this work and should be further investigated. The comparison of the error detection sensitivity between the H&N and prostate localization showed a weaker detection of errors for the prostate localization. This trend is explained by the spherical shape of the target volume. As a result, the collimator errors have less impact and are therefore less detected. This trend was also found by Fredh et al. as well as Vieillevigne et al.^11^, ^13^ In our study, the detection sensitivity of MLC errors was similar regardless of localization and measurement process. The slight difference for dose errors can be explained by the fact that the modulation is less strong for prostate plans. Regardless of the measurement process and the localization, the 3%/3 mm/Global analysis protocol had the lowest detection sensitivity varying from 54% for AC to 40% for PDIP, for H&N plans and 30% for AC to 16% for PDIP, for prostate plans (Tables 2 and 3). This result confirms the study of Heileman et al. which indicated that this protocol was not stringent enough to evaluate treatment plan quality.^12^ In this study, the 3%/3 mm/Local analysis protocol was more effective regardless of the process studied. For the PDIP, only this analysis protocol allowed the early detection of simulated errors. The impact of the normalization mode and the thresholding criterion should be investigated. However, the mean pass rate of ref H&N plans showed a risk of false positive for AC (95.6% ± 2.6%). A statistical study on a larger sample will be necessary to ensure that the treatment plans in clinical routine respect the PR.

Compared to single process, the combined use of AC and PDIP which is more time‐consuming in clinical routine did not significantly improve the error detection sensitivity. In our study, the combined process increased the total error detection sensitivity of AC from +0% to +4%, depending on analysis protocol and localization. This result is in agreement with Fredh et al. who showed a marginal benefit in the use of multiple detectors.^13^ Moreover, none of the processes (AC, PDIP, or combined) allowed systematic detection of all errors. This result is in agreement with the study of Kry et al. which highlighted the low power of pretreatment QC.^16^

Finally, the individual use of the AC or the PDIP with an optimal analysis protocol made it possible to obtain an error detection sensitivity equal to or greater than combined measurement process. For example, the individual use of AC or PDIP with 3%/3 mm/Local analysis protocol had an error detection sensitivity equal to or greater than the combined process with 3%/3 mm/Global analysis protocol (Tables 2 and 3).

This study highlights the importance of optimizing the analysis protocols, particularly according to the localization and to the measurement process in order to find the right balance between error detection sensitivity and false positive risk.

## 5 CONCLUSION

This work showed that the combined use of AC and PDIP did not significantly improve the error detection sensitivity compared to use of a single process. None of the measurement processes used individually or in combination allowed systematic detection of all errors. The analysis protocols optimization of each measurement process appeared necessary in order to obtain an optimal error detection sensitivity.

## CONFLICTS OF INTEREST

The authors have no relevant conflicts of interest to disclose.
